# Cell signaling dependence of rapid glucocorticoid-induced endocannabinoid synthesis in hypothalamic neuroendocrine cells

**DOI:** 10.1016/j.ynstr.2019.100158

**Published:** 2019-03-21

**Authors:** Christina Harris, Grant L. Weiss, Shi Di, Jeffrey G. Tasker

**Affiliations:** a Department of Cell and Molecular Biology, Tulane University, New Orleans, LA, USA; 2 Tulane Brain Institute, Tulane University, New Orleans, LA, USA

## Abstract

Glucocorticoids induce a rapid synthesis of endocannabinoid in hypothalamic neuroendocrine cells by activation of a putative membrane receptor. Somato-dendritically released endocannabinoid acts as a retrograde messenger to suppress excitatory synaptic inputs to corticotropin-releasing hormone-, oxytocin-, and vasopressin-secreting cells. The non-genomic signaling mechanism responsible for rapid endocannabinoid synthesis by glucocorticoids has yet to be fully characterized. Here we manipulated cell signaling molecules pharmacologically using an intracellular approach to elucidate the signaling pathway activated by the membrane glucocorticoid receptor in hypothalamic neuroendocrine cells. We found that rapid glucocorticoid-induced endocannabinoid synthesis in magnocellular neuroendocrine cells requires the sequential activation of multiple kinases, phospholipase C, and intracellular calcium mobilization. While there remain gaps in our understanding, our findings reveal many of the critical players in the rapid glucocorticoid signaling that culminates in the retrograde endocannabinoid modulation of excitatory synaptic transmission.

## 1 Introduction

Endocannabinoids are endogenous analogues of the plant-derived cannabinoid Δ^9^ tetrahydrocannabinol that act at cannabinoid receptors. The two major endocannabinoids, anandamide (arachidonoylethanolamine, AEA) and 2-arachidonoylglycerol (2-AG) are endogenous ligands for the type I and type 2 cannabinoid (CB1 and CB2) receptors (Herkenham et al., 1990). The CB1 receptors are widely expressed throughout the brain and spinal cord and support various forms of short- and long-term synaptic plasticity in virtually every brain region studied to date (Kano et al., 2009). In the hypothalamic paraventricular nucleus (PVN), for example, endocannabinoids are involved in the milk ejection reflex (Rossoni et al., 2008), in fluid and electrolyte homeostasis (Kuzmiski et al., 2009), and in the negative feedback regulation of the hypothalamic pituitary adrenal (HPA) axis (Barna et al., 2004; Cota, 2007; Di et al., 2003; Evanson et al., 2010; Hill et al., 2010). CB1 receptors are localized at the presynaptic terminals of many excitatory and inhibitory synapses, where they suppress glutamate and GABA release (Freund et al., 2003). Endocannabinoids can be produced transiently by strong postsynaptic depolarization and calcium influx through voltage-gated calcium channels (Maejima et al., 2001; Ohno-Shosaku et al., 2002) and in response to synaptic activity (Azad et al., 2004; Chevaleyre and Castillo, 2003; Chiu et al., 2010; Di et al., 2005a) and G protein-coupled receptor activation linked to phospholipase C (PLC) signaling (Kano et al., 2009; Maejima et al., 2001; Varma et al., 2001). In many instances, group 1 metabotropic glutamate receptors or muscarinic acetylcholine receptors linked to PLC converge with calcium signaling mechanisms to decrease the threshold for endocannabinoid synthesis (Hashimotodani et al., 2005; Ohno-Shosaku et al., 2002). Endocannabinoid synthesis has also been reported to require the activity of protein kinases. Specifically, cAMP-dependent protein kinase (PKA) and protein kinase C (PKC) have both been implicated in endocannabinoid production (Cadas et al., 1996; De Petrocellis et al., 2008), however the specific role of kinase activity in endocannabinoid release has yet to be fully elucidated.

We have reported that activation of a membrane-associated glucocorticoid receptor rapidly suppresses excitatory synaptic transmission in parvocellular and magnocellular neuroendocrine cells of the hypothalamic PVN and supraoptic nucleus (SON) by inducing the synthesis and retrograde transmission of endocannabinoids through a G protein-dependent mechanism (Di et al., 2003; Malcher-Lopes et al., 2006) (Di et al., 2005a). Previously, we found the rapid glucocorticoid-induced endocannabinoid synthesis to be dependent on the activation of a Gs/cAMP/PKA signaling pathway (Malcher-Lopes et al., 2006) and on the nuclear glucocorticoid receptor (Nahar et al., 2015). In addition to PKA, PKC signaling was also implicated in the rapid glucocorticoid-induced endocannabinoid signaling (Di et al., 2003), but it was not determined whether the site of PKC activity is postsynaptic, downstream from the glucocorticoid receptor, or presynaptic, in the CB1 receptor signaling pathway. In magnocellular neurosecretory neurons, postsynaptic oxytocin receptors, which are coupled to PKC and calcium signaling (Irani et al., 2010), induced retrograde endocannabinoid release in the SON (Hirasawa et al., 2004). Interestingly, endocannabinoid release occurred in the absence of action potentials, suggesting that voltage-gated calcium channels are not involved.

In this study we used whole-cell patch clamp recordings and intracellular and extracellular pharmacological manipulations to probe the sequential kinase and calcium signaling mechanisms responsible for the rapid glucocorticoid-induced endocannabinoid modulation of excitatory synaptic transmission. Recordings were performed in magnocellular neuroendocrine cells of the PVN and SON as a model of rapid glucocorticoid modulation of neuroendocrine cells, since both PVN magnocellular and parvocellular neuroendocrine cells respond to glucocorticoids with a rapid induction of retrograde endocannabinoid signaling at excitatory synapses (Di et al., 2005a, 2003). We did not distinguish between magnocellular oxytocin and vasopressin neurons in this study because both cell types respond in a similar fashion to glucocorticoids (Di et al., 2009, 2005b).

## 2 Methods

### 2.1 Brain slice preparation

Male Sprague Dawley rats (4–6 weeks old, Charles River, Wilmington, MA) were used in most of these experiments; one experiment was done in neurons from older male Sprague Dawley rats (9–11 weeks) for confirmation of our findings in adult subjects. All experiments were performed in accordance with a protocol approved by the Tulane University Institutional Animal Care and Use Committee. Hypothalamic slices containing the SON or PVN were prepared as described previously (Di et al., 2003). Briefly, rats were anesthetized with isoflurane, decapitated, and the brain was quickly removed and submerged in an ice-cooled, oxygenated (100% O_2_) artificial cerebral spinal fluid (aCSF) containing (in mM): 140 NaCl, 3 KCl, 1.3 MgSO_4_, 1.4 NaH_2_PO_4_, 2.4 CaCl_2_, 11 glucose, and 5 HEPES. The hypothalamus was blocked and coronal slices containing the PVN and SON were sectioned at 300–350 μm in thickness on a vibratome (Leica, Wetzlar, Germany). The slices were maintained in a holding chamber containing oxygenated aCSF at room temperature for ≥1.5 h before being transferred to a submersion recording chamber on a fixed-stage upright microscope (Olympus BX50WI). There, the slices were perfused with oxygenated aCSF and individual cells were visualized using infrared illumination and differential interference contrast optics.

### 2.2 Whole-cell recordings

Whole-cell patch clamp recordings were performed at 32–34 °C using electrodes formed on a horizontal puller (P-97, Sutter Instruments, Sacramento, CA) with a tip resistance of 3.5–4.5 MΩ. Electrodes were filled with an internal solution containing (in mM): 120 K-gluconate, 10 KCl, 1 NaCl, 1 MgCl_2_, 1 CaCl_2,_ 10 EGTA, 2 Mg-ATP, 0.3 Na-GTP, and 10 HEPES. All recordings were performed in voltage-clamp mode using a Multiclamp 700A amplifier and pCLAMP 9 software (Molecular Devices, Sunnyvale, CA). Data were low-pass filtered at 2 kHz and digitized at 5–10 kHz. Magnocellular neurons in the PVN and the SON were targeted based on morphology, which was confirmed by the presence of an A-type potassium current (Luther and Tasker, 2000). To record miniature excitatory postsynaptic currents (mEPSCs), the magnocellular neurons were voltage clamped at a holding potential of −60 mV, tetrodotoxin (TTX, 1 μM) was added to the aCSF to block spike-mediated transmitter release, and picrotoxin (50 μM), a GABA_A_ receptor antagonist, was added to block inhibitory postsynaptic currents. All recordings were allowed to stabilize for 5–10 min prior to the start of pharmacological experiments. Input resistance and series resistance were monitored for stability during the recordings.

### 2.3 Drug application

Table 1 shows the drugs used, their targets, and their working concentrations. Bath-applied drugs were stored as aqueous stock solutions at −20 °C and were dissolved in aCSF to their final concentrations just prior to their application in the bath perfusion. These included the water-soluble glucocorticoid (2-hydroxypropyl)-β-cyclodextrin-conjugated dexamethasone (Dex), the voltage-gated sodium channel inhibitor tetrodotoxin (TTX), the Ca^2+^-ATPase inhibitor thapsigargin, the inositol triphosphate 3 (IP3) receptor inhibitor XestosponginC, the proto-oncogene tyrosine-protein kinase Src (Src) inhibitor 1-(1,1-dimethylethyl)-3-(4-methylphenyl)-1H-pyrazolo[3,4-d]pyrimidin-4-amine (PP1), and the CB1 synthetic agonist WIN55,212. Drugs applied intracellularly were added to the electrode solutions at their final concentrations on the day of experiments. These included the PKA activator 8-Br-cAMP, the PKC activator SC-10, and the calcium chelator 1,2-Bis(2-aminophenoxy)ethane-*N*,*N*,*N*′,*N*′-tetraacetic acid (BAPTA). The CB1 antagonist AM251 was made directly in aCSF on the day of experiments. All drugs were purchased from either Sigma-Aldrich (St. Louis, MO) or Tocris Cookson Inc. (Ellisville, MO) except DO34, which was a generous gift from Benjamin Cravatt (Ogasawara et al., 2016).

**Table 1:** **Drug concentrations and Targets**. The following references were used to determine the drug concentrations used: (a) (Di et al., 2003), (b) (Sharon et al., 1997), (c) (Ogasawara et al., 2016), (d) (Davis et al., 2003), (e) (Ricciardi et al., 2005), (f) (Khomula et al., 2017), (g) (Gnegy et al., 2004), (h) (Kotturi et al., 2003), (i) (Hoffman and Johnston, 1998), (j) (Iannazzo et al., 1999), (k) (Jin et al., 2009), (l) (Liu et al., 2018), (m) (Bisogno et al., 2006).

**Drug**	**Target**	**Conc.(Ref.)**	**Source**
**Extracellular application**			
Dex-cyclodextrin	GR	1 μM(a)	Sigma
WIN 55,212-2	CB1 agonist	5 μM(a)	Tocris
AM251	CB1 antagonist	4 μM(a)	Sigma
Bis(indolylmaleimide)	PKC inhibitor	1 μM(b)	Sigma
DO34	DAGL Inhibitor	1 μM(c)	Cravatt Lab
U0126	ERK inhibitor	10 μM(d)	Sigma
PD 0325901	ERK inhibitor	100 nM(e)	Tocris
Xestospongin C	IP3R antagonist	1 μM(f)	Sigma
Thapsigargin	ER Ca ATPase	5 μM(g)	Sigma
THL	Lipase inhibitor	25 μM(m)	Sigma
Nifedipine	V-gated Ca2+ block	10 μM(h)	Sigma
PP1	SRC inhibitor	10 μM(d)	Tocris
**Intracellular application**			
8-Br-cAMP	PKA activator	100 μM(i)	Sigma
SC-10	PKC activator	500 nM(j)	Sigma
PKC19-31	PKC inhibitor	10 μM(k)	Sigma
BAPTA	Ca2+ chelator	40 mM(g)	Sigma
m3M3FBS	PLC activator	10 μM(l)	Tocris

### 2.4 Data analysis

To study the effects of drugs on mEPSCs, 3-min episodes of baseline mEPSC activity were collected just prior to drug application and compared to 3-min episodes at the end of a 10-min application of the drug. mEPSCs were analyzed for changes in mean frequency, peak amplitude, and decay time (defined as the time from peak to 63% decay) using the Minianalysis 6.0 program (Synaptosoft Inc., Decatur, GA). Statistical analysis was performed with Prism 7.0 (GraphPad, La Jolla, CA) using a two-tailed Student's paired *t*-test (drug vs. baseline), unless otherwise noted. An ANOVA was performed when more than two groups were simultaneously compared, followed by a Bonferroni *post-hoc* analysis. Probability values < 0.05 were considered significant. The numbers of replicates provided for each experiment refer to the numbers of magnocellular neurons recorded; each experiment was performed in brain slices from at least 3 different animals.

## 3 Results

### 3.1 Glucocorticoids rapidly suppress excitatory synaptic inputs via 2-AG release

**Fig. 1:**
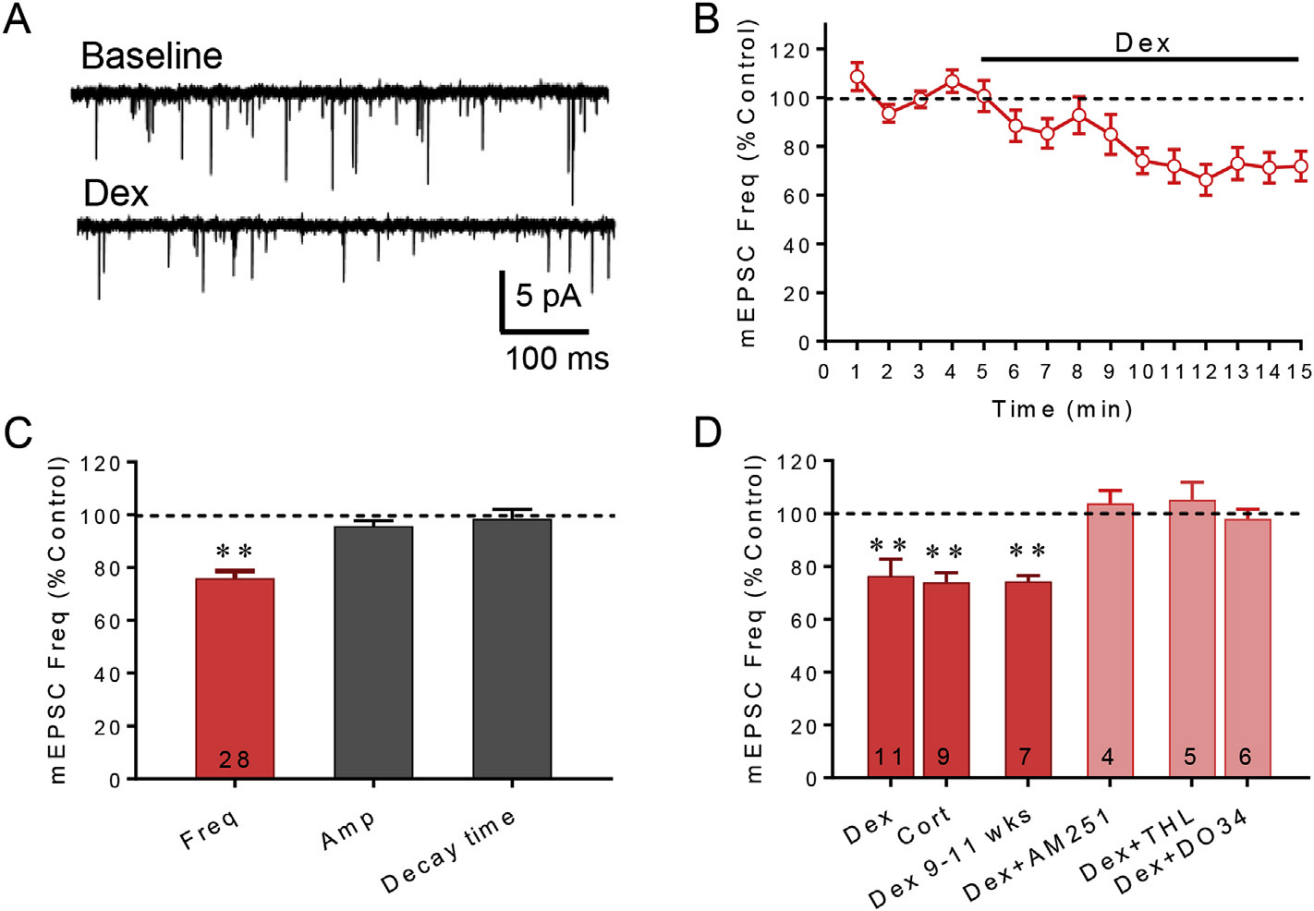
**Glucocorticoids rapidly suppress excitatory synaptic inputs to magnocellular neurons by causing 2-AG release.** A. Representative traces showing the effect of bath application of the synthetic glucocorticoid agonist dexamethasone (Dex) on mEPSCs. B. Running average of mEPSC frequency indicates that the Dex effect is rapid, occurring within minutes (n = 11). C. Summary bar graph of effect of Dex (1 μM) on mEPSC frequency, amplitude, and decay time. Dex decreased the mEPSC frequency but had no effect on the mEPSC amplitude or decay, indicating a presynaptic effect. D. Corticosterone (Cort, 1 μM) also caused a decrease in the frequency of mEPSCs. The Dex-dependent decrease in mEPSC frequency was found in magnocellular neurons from both 4-6 week-old (Dex) and 9-11 week-old (Dex 9–11 wks, p < 0.01, n = 7) animals, and was blocked by the CB1 antagonist AM251 (4 μM, *n* = 4) and the DAG lipase inhibitors tetrahydrolipstatin (THL, 25 μM, *n* = 5) and DO34 (1 μM, n = 7). Numerals in bars = cell numbers in this and all figures. *, *p* < 0.05; **, *p* < 0.01.

Previously, we demonstrated that the glucocorticoids dexamethasone (Dex) and corticosterone (Cort) cause a significant decrease in the frequency of mEPSCs in magnocellular and parvocellular neuroendocrine cells of the PVN and SON by stimulating the synthesis and release of a retrograde endocannabinoid messenger (Di et al., 2003; Malcher-Lopes et al., 2006). Here, Dex (1 μM) applied in the bath perfusion caused a ∼24% average decrease in the mEPSC frequency in all PVN and SON magnocellular neurons tested (*p* < 0.05, n = 11) (Fig. 1A–C). The Dex-dependent decrease in mEPSC frequency had a rapid onset (Fig. 1B), indicating a non-genomic mechanism of glucocorticoid action. Dex had no effect on the amplitude or decay time of mEPSCs (Fig. 1C), which suggested a presynaptic mechanism. The endogenous adrenal corticosteroid corticosterone (Cort, 1 μM, n = 9) caused a similar 25% decrease in the mEPSC frequency (Fig. 1D). The effect of Dex on mEPSC frequency was mediated by endocannabinoid activation of CB1 receptors, since it was blocked by the selective CB1 receptor inverse agonist AM251 (4 μM) (*p* = 0.98, *n* = 4) (Fig. 1D). The Dex suppression of mEPSC's was also found in 9–11 week-old rats (p < 0.01, n = 6) (Fig. 1D), confirming that glucocorticoids also mobilize endocannabinoids in adult animals.

While the AEA synthesis pathway has not yet been well characterized in neurons (Simon and Cravatt, 2008), 2-AG synthesis has been shown to be a product of diacylglycerol lipase (DAGL) activity (Ahn et al., 2008; Yoshino et al., 2011). Therefore, we targeted 2-AG synthesis using two specific blockers of DAGL, tetrahydrolipstatin (THL) (Bisogno et al., 2006) and DO34 (Ogasawara et al., 2016). THL (25 μM) was bath applied for 10 min, followed by the co-application of Dex (1 μM) and THL (25 μM). In the presence of THL, the Dex-induced decrease in mEPSC frequency was abolished (*p* = 0.53, *n* = 5) (Fig. 1D). Similarly, DO34 (1 μM, 30 min pre-incubation) also blocked the Dex (1 μM)-induced decrease in mEPSC frequency (p = 0.60, n = 7) (Fig. 1D). A between-group comparison revealed significant differences between the effects on mEPSC frequency of Dex alone and Dex in the presence of THL (*p* < 0.01) and DO34 (p < 0.01).

### 3.2 Glucocorticoid-induced endocannabinoid release is PKC dependent

**Fig. 2:**
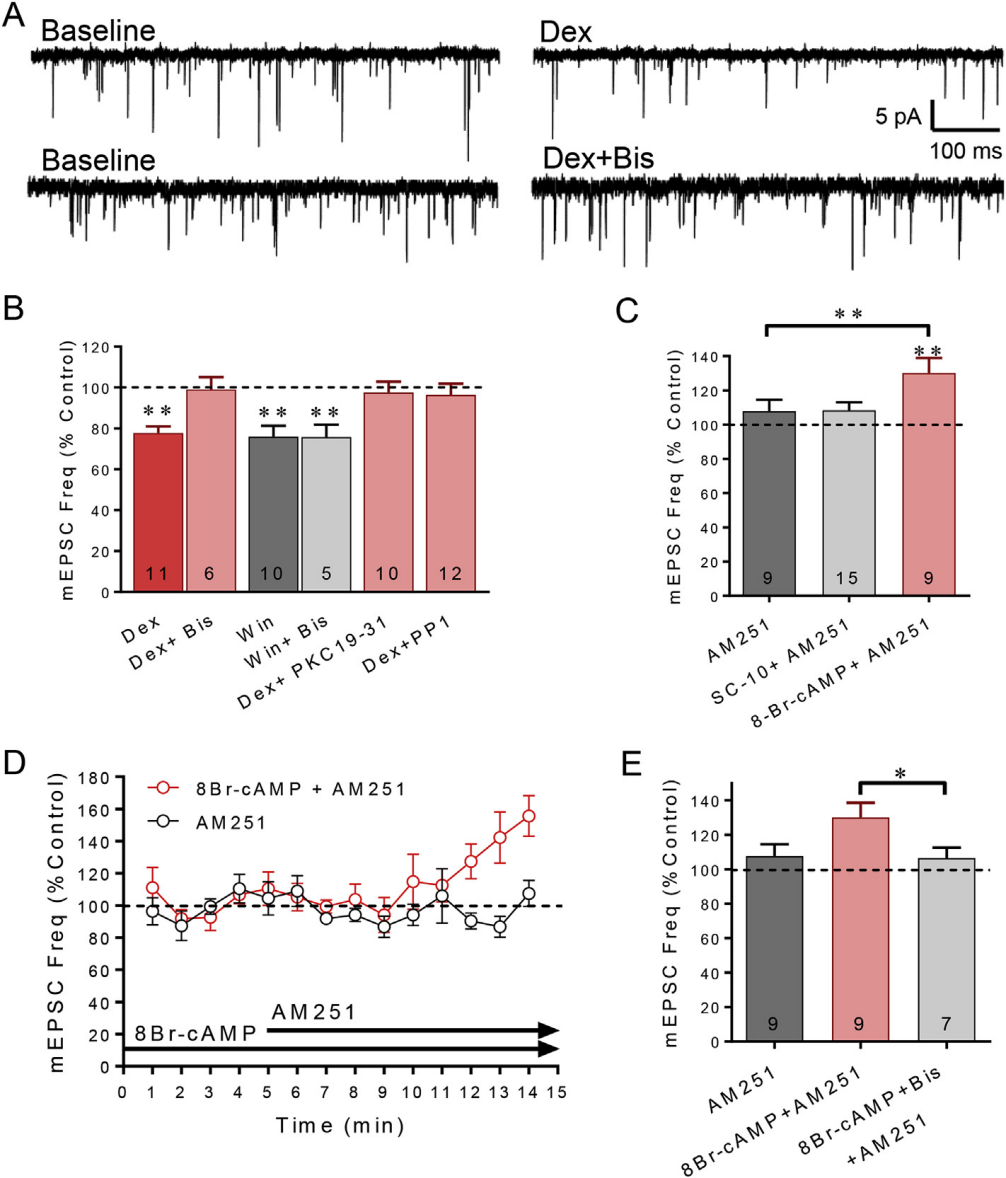
**PKC, PKA, and Src signaling in the rapid glucocorticoid-induced suppression of excitation.** A. Representative traces of the effect of dexamethasone on mEPSC frequency compared to baseline in the absence (Dex) and presence of the broad-spectrum, membrane-permeant PKC inhibitor bisindolylmaleimide (Dex + Bis). B. Bath application of Bis (Dex + Bis) (n = 6) blocked the Dex-induced decrease in mEPSC frequency but did not block the decrease in mEPSC frequency elicited by bath application of the CB1 receptor agonist Win55,212 (baseline vs. Win + Bis, *p* < 0.01; Win vs. Win + Bis, *p* = 0.68). The Dex-dependent decrease in mEPSC frequency was also prevented by intracellular application of the peptide PKC inhibitor (Dex + PKC19-31 vs. Dex) (*p* < 0.01). Bath application of the Src inhibitor PP1 blocked the Dex-induced suppression of mEPSC frequency (Dex + PP1). C. The CB1 receptor antagonist AM251 had no effect on mEPSC frequency alone, indicating that there was not a baseline tonic release of endocannabinoid at glutamate synapses. Following intracellular application of the PKC activator SC-10 (50 μM) via the patch electrode, AM251 had no effect on mEPSC frequency, suggesting that PKC activation did not cause endocannabinoid release. Following intracellular application of the PKA activator 8-Br-cAMP (100 μM) via the patch electrode, AM251 caused a significant increase in mEPSC frequency, suggesting that PKA activation caused an endocannabinoid-dependent suppression of mEPSCs. D. Running average of mEPSC frequency with continuous intracellular application of the PKA activator 8-Br-cAMPs via the patch pipette (100 μM) and following bath application of the CB1 receptor antagonist AM251 (cAMP + AM251, 4 μM). AM251 caused an increase in mEPSC frequency in recordings in which 8-Br-cAMP was included in the patch electrode. E. Summary bar graph of the mean effects of PKA activation and CB1 receptor and PKC inhibition on normalized mEPSC frequency. Blocking CB1 receptors alone had no effect on mEPSC frequency (AM251) but caused an increase in mEPSC frequency following intracellular activation of PKA (cAMP + AM251), suggesting that postsynaptic PKA activation caused endocannabinoid release. Blocking PKC activity with bath application of bisindolylmaleimide blocked the AM251-induced increase in mEPSC frequency in neurons recorded with 8BR-cAMP in the electrode (cAMP + Bis + AM251), suggesting that the postsynaptic PKA activation of endocannabinoid release was PKC-dependent. *, *p* < 0.05; **, *p* < 0.01.

In previous studies, we found that the rapid glucocorticoid-induced suppression of glutamate release was abolished in the presence of a PKC inhibitor applied to the bath (Di et al., 2005a, 2003). However, this did not allow us to distinguish between a PKC signaling mechanism in the postsynaptic glucocorticoid receptor signaling pathway or in the presynaptic CB1 receptor signaling pathway. Here, we confirmed the PKC dependence of the rapid glucocorticoid effect with the broad-spectrum PKC inhibitor bisindolylmaleimide (GF109203X, Bis). Bath application of Dex (1 μM, n = 11) caused a 24% decrease in the frequency of mEPSCs, (*p* < 0.01) (Fig. 2A and B). A 10-min bath application of Bis alone (1 μM) had no effect on mEPSC frequency (*p* = 0.22, n = 12), but abolished the rapid Dex-induced decrease in mEPSC frequency (*p* = 0.48, *n* = 6) (Fig. 2A and B). A between-group comparison revealed a significant difference between the effect of Dex in the absence and in the presence of bis (*p* < 0.01; unpaired *t*-test).

Since the PKC inhibitor is applied to the bath and the presynaptic CB1 receptor could also be coupled to a PKC signaling cascade (Bosier et al., 2008; Garcia et al., 1998), we tested for the possible attenuation of presynaptic CB1 receptor signaling by the PKC inhibitor. Bath application of the membrane-permeant PKC inhibitor Bis (1 μM) for 10 min was followed by the co-application of Bis and the synthetic CB1 receptor agonist Win55,212-2 (5 μM). The PKC inhibitor failed to block the decrease in mEPSC frequency elicited by CB1 receptor activation with WIN55,212-2. Win55,212-2 alone caused a 25% decrease in mEPSC frequency, and Win55,212-2 in the presence of Bis caused a 26% decrease in mEPSC frequency (*p* = 0.68, unpaired *t*-test, n = 10 and 5 respectively) (Fig. 2B), indicating that the presynaptic signaling by the CB1 receptor is not PKC dependent and that the PKC dependence of the glucocorticoid effect is in the postsynaptic membrane glucocorticoid receptor signaling pathway.

Next, to further test whether the PKC dependence of the rapid glucocorticoid effect resides in the postsynaptic membrane glucocorticoid receptor signaling pathway, we applied a membrane-impermeant peptide PKC inhibitor, PKC 19–31 (10 μM), directly into the postsynaptic cell via the patch pipette. Following a 10–15 min period of intracellular PKC 19–31 infusion after breaking into the cell, bath application of Dex (1 μM) had no effect on the mEPSC frequency (*p* = 0.32, *n* = 10) (Fig. 2B). A between-cell comparison revealed a significant difference in the effect of Dex alone versus Dex in the presence of the intracellular PKC inhibitor (*p* < 0.01; unpaired *t*-test) (Fig. 2B). Bath application of Cort also failed to elicit a reduction in mEPSC frequency following intracellular PKC 19–31 (10 μM) application (94% of baseline, p = 0.17, n = 6). The effect of Cort alone was significantly different than the effect of Cort in the presence of the intracellular PKC inhibitor in a between-cell comparison (*p* < 0.01; unpaired *t*-test). These data together indicate that the membrane glucocorticoid receptor signaling that leads to retrograde endocannabinoid release is dependent on PKC activity.

### 3.3 PKA activation is sufficient for endocannabinoid synthesis

We showed previously that rapid glucocorticoid-induced endocannabinoid synthesis is also dependent on a Gs, cAMP and cAMP-dependent protein kinase (PKA) signaling pathway (Malcher-Lopes et al., 2006). To determine whether PKC and PKA activation is sufficient for endocannabinoid production independent of glucocorticoid receptor activation, the PKA activators SpcAMPs (10 μM) and 8-Bromo cAMP (100 μM) and the PKC activator SC-10 (0.5 μM) were applied intracellularly via the patch pipette and the recordings were analyzed for changes in mEPSC frequency. This method of kinase activation by intracellular infusion did not allow a pre-drug period of baseline mEPSC measurement prior to the drug taking effect. Therefore, we assayed for presynaptic CB1 receptor activation by bath application of the CB1 antagonist AM251 after >10 min of intracellular infusion of the kinase activators. AM251 (4 μM) alone (i.e., without intracellular kinase activator) had no significant effect on mEPSC frequency (*p* = 0.52, *n* = 8), indicating there was no tonic or constitutive CB1 receptor activation at glutamate synapses (Fig. 2C). Following intracellular infusion of the PKC activator SC-10 (0.5 μM) for >10 min, bath application of AM251 (4 μM) had no effect on mEPSC frequency (*p* = 0.65, *n* = 15) (Fig. 2C). In contrast, following intracellular infusion of the PKA activator 8-Bromo cAMP (100 μM) for > 10 min, bath application of AM251 (4 μM) caused a 30% increase in mEPSC frequency in magnocellular neurons (*p* < 0.05, *n* = 9) (Fig. 2C). These results suggest that PKA is both necessary and sufficient for glucocorticoid-induced endocannabinoid release, while PKC activity is necessary for the glucocorticoid-induced endocannabinoid modulation but is not sufficient alone to induce the retrograde release of endocannabinoid.

Src is a tyrosine kinase that has been shown to be involved in both PKA- and PKC-dependent pathways stimulated by corticosterone in the hippocampus (Yang et al., 2013). Furthermore, the nuclear glucocorticoid receptor (GR) complexes with Src in a ligand-dependent manner (Leo and Chen, 2000). Therefore, we tested for Src dependence using the Src inhibitor PP1. In slices preincubated with PP1 (10 μM) for > 60 min, the Dex failed to induce a decrease in mEPSC frequency (Fig. 2B), signifying a dependence of the glucocorticoid-induced endocannabinoid synthesis on Src activity.

### 3.4 PKC is downstream from PKA in the membrane glucocorticoid receptor signaling pathway

In order to determine the sequence of PKA and PKC activation in the membrane glucocorticoid receptor signaling pathway, we applied a strategy combining activators and inhibitors of the different kinases. 8-Br-cAMP (100 μM) was applied intracellularly via the patch pipette to activate PKA activity, followed by the bath application of either the CB1 receptor antagonist AM251 (4 μM) alone or AM251 and the PKC antagonist Bis (1 μM) to test whether blocking PKC activity blocks the PKA-induced endocannabinoid retrograde signaling. With 8-Br-cAMP applied intracellularly, the AM251-induced increase in mEPSC frequency was blocked in the presence of the PKC inhibitor Bis (106% of baseline, *p* = 0.31, *n* = 7) (Fig. 2D and E), indicating that inhibiting PKC abolishes the PKA-induced endocannabinoid effect and that PKC signaling, therefore, is downstream of, and necessary for, PKA-induced endocannabinoid synthesis. The PKC dependence was in the membrane glucocorticoid receptor signaling pathway because bath application of the PKC inhibitor had no effect on CB1 receptor signaling (see Fig. 2B).

### 3.5 Glucocorticoid-induced endocannabinoid release is dependent on ERK activation

**Fig. 3:**
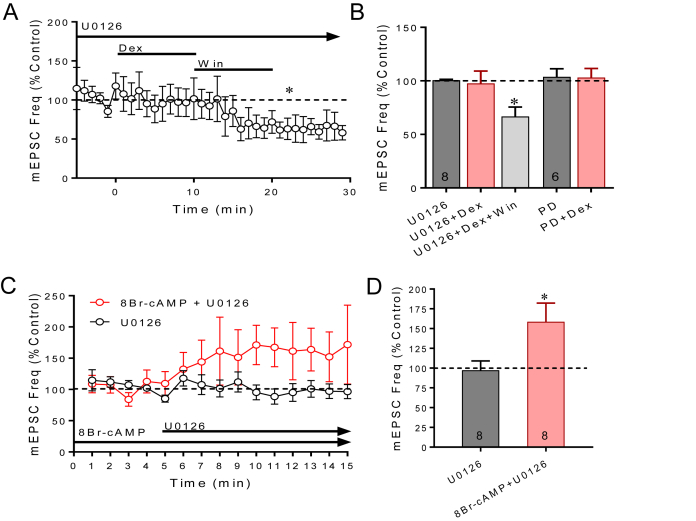
**ERK-MAP kinase activation is required for glucocorticoid-induced suppression of excitation.** A. Running average of mEPSC frequency in the presence in the bath perfusion of the MEK inhibitor U0126 (25 μM). U0126 blocked the effect of Dex (1 μM) on the frequency of mEPSCs but did not block the decrease in the mEPSC frequency caused by activation of presynaptic CB1 receptors with Win55,212-2 (Win, 5 μM), suggesting ERK/MAPK dependence of glucocorticoid signaling. B. Summary bar graph of the effect of the MEK inhibitors U0126 and PD 0325901 on the Dex-induced suppression of mEPSCs. The MEK inhibitors blocked the suppression of mEPSC frequency by Dex but not by the CB1 agonist WIN55,212-2. C. The PKA-induced endocannabinoid suppression of mEPSCs is dependent on ERK-MAPK activity. The MEK inhibitor U0126 applied alone had no effect on mEPSC frequency but caused an increase in mEPSC frequency following the intracellular application of the PKA activator 8Br-cAMP via the patch pipette. D. Summary bar graph of mean effects of blocking ERK-MAPK activity with U0126 in the absence and presence of the PKA activator 8-Br-cAMP in the patch pipette (cAMP + U0126). *, *p* < 0.05.

The ERK-MAPK signaling pathway is canonically downstream of PKC and phospholipase C (PLC) and could also be required for endocannabinoid synthesis. We tested for ERK-MAPK dependence of the rapid glucocorticoid-induced endocannabinoid modulation using two MEK inhibitors, U0126 and PD 0325901. Bath application of U0126 (10 μM) blocked the Dex-induced decrease in mEPSC frequency (1 μM, 10 min) (Fig. 3A and B), but not the decrease in the mEPSC frequency induced by the CB1 agonist WIN 55,212-2 (1 μM) (repeated measures ANVOA, p < 0.05; *post-hoc* WIN vs U0126, p < 0.05, n = 8), indicating that ERK/MAPK signaling is in the postsynaptic membrane glucocorticoid receptor pathway. We also applied the membrane-impermeant, selective MEK inhibitor PD 0325901 (PD, 100 nM) directly into the postsynaptic cell via the patch pipette, followed > 5 min later by the bath application of Dex (1 μM). Dex had no effect on the mEPSC frequency when MEK activity was blocked specifically in the postsynaptic cell (p < 0.05, unpaired *t*-test vs Dex) (Fig. 3B), which confirmed the postsynaptic ERK/MAPK dependence of the rapid glucocorticoid-induced endocannabinoid release.

Using the same strategy we used to determine whether PKC is downstream from PKA, we sequentially activated PKA signaling before blocking ERK-MAPK activity to determine if ERK signaling is also downstream of PKA in the membrane glucocorticoid receptor signaling pathway. The PKA activator 8-Br-cAMP was applied intracellularly via the patch pipette, followed by the bath application of the MEK inhibitor U0126 (10 μM). The MEK inhibitor alone, without the intracellular PKA activator, had no effect on the mEPSC frequency, whereas it caused a 61% increase in mEPSC frequency following the intracellular application of the PKA activator (p < 0.05, n = 8) (Fig. 3C and D), indicating a block of the PKA-induced suppression of synaptic excitation, which we showed was dependent on CB1 receptor activation (see Fig. 2C). The PKA stimulation of endocannabinoid synthesis in magnocellular neurons, therefore, requires ERK-MAPK activation. These findings together, therefore, indicate that PKA, PKC and ERK-MAPK activities are necessary for the membrane glucocorticoid receptor activation of 2-AG synthesis, and that PKC and ERK-MAPK are downstream from PKA activation in the membrane glucocorticoid receptor pathway.

### 3.6 Glucocorticoid-induced endocannabinoid release requires postsynaptic calcium

**Fig. 4:**
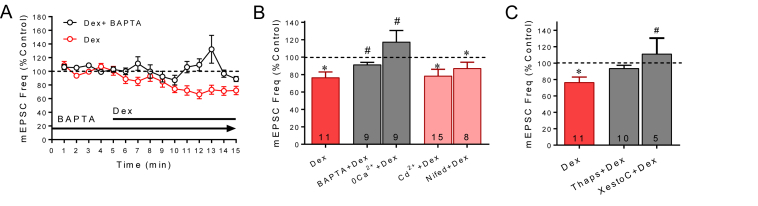
**Glucocorticoid-induced suppression of excitation requires postsynaptic calcium mobilization.** A. Running time histogram of the effect on mEPSC frequency of Dex with and without BAPTA in the patch electrode. The Dex-induced decrease in mEPSC frequency was blocked with BAPTA in the electrode. B. Summary bar graph of the effects of calcium blockers on the Dex-induced decrease in mean normalized mEPSC frequency. Dexamethasone had no effect on the mEPSC frequency following intracellular application of BAPTA or extracellular application of a nominally zero calcium solution (0Ca^2+^) but retained its suppressive effect on mEPSC frequency in cells treated with the voltage-gated calcium channel blockers cadmium (Cd^2+^, 200 μM) and nifedipine (Nifed, 10 μM) applied in the perfusion bath. C. Summary bar graph of the effects of blockers of intracellular calcium mobilization. Inhibition of the SERCA intracellular calcium transporter with intracellular thapsigargin application via the patch pipette blocked the Dex-induced suppression of mEPSC's (Thaps, 5 μM). Inhibition of the IP3 receptor antagonist Xestospongin C also blocked the Dex-induced decrease in mEPSC frequency (XestoC). *, *p* < 0.05 compared to baseline; **, *p* < 0.01 compared to baseline; #, *p* < 0.05 vs Dex).

Endocannabinoid release is often dependent on calcium signaling (Maejima et al., 2001; Zampronio et al., 2010). Therefore, we sought to determine whether the rapid glucocorticoid-induced endocannabinoid release from magnocellular neurons is calcium dependent. The membrane-impermeant, fast calcium chelator BAPTA (40 mM) was applied intracellularly via the patch pipette to block calcium signaling. Following establishment of the whole-cell configuration and infusion of BAPTA into the postsynaptic cell for 20 min, Dex (1 μM) was bath-applied for 10 min. The rapid Dex-dependent decrease in mEPSC frequency was not seen in recordings with BAPTA in the patch electrodes (*p* = 0.11, *n* = 8) (Fig. 4A and B). A between-group comparison revealed a significant difference in the Dex effect between recordings performed with and without BAPTA in the patch electrodes (*p* < 0.01, unpaired *t*-test). Therefore, the rapid glucocorticoid-induced endocannabinoid suppression of excitation requires postsynaptic calcium signaling.

2-AG release is induced by calcium influx in cortical and cerebellar neurons (Hashimotodani et al., 2005; Ohno-Shosaku et al., 2002; Stella and Piomelli, 2001). To determine if 2-AG production in neuroendocrine cells is dependent on extracellular calcium, we used an extracellular solution with no calcium and with the calcium chelator EGTA (0.2–200 mM), which we refer to as a 0-Ca solution. Bath application of the 0-Ca solution had no significant effect alone on the frequency of mEPSCs (*p* = 0.36, *n* = 9), but abolished the Dex-induced decrease in mEPSC frequency (*p* = 0.27, *n* = 9) (Fig. 4B), suggesting that the glucocorticoid-induced endocannabinoid synthesis is dependent on extracellular calcium. A between-group comparison of the effects of Dex in the absence and presence of the 0-Ca solution revealed a significant difference between the two groups (*p* < 0.05, unpaired *t*-test).

We next tested for the dependence of the rapid glucocorticoid effect on calcium influx through voltage-gated calcium channels using the broad-spectrum calcium channel blocker cadmium and the L-type calcium channel blocker nifedipine. Cadmium (200 μM) and nifedipine (10 μM) applied in the bath had no effect alone on mEPSC frequency (cadmium: *p* = 0.64, *n* = 15; nifedipine: *p* = 0.57, *n* = 8). Neither of the two calcium channel antagonists had any effect on the Dex-induced decrease in mEPSC frequency (*p* < 0.05, *n* = 15 and *n* = 8, respectively) (Fig. 4B). There was no significant difference in the Dex-induced decrease in mEPSC frequency in cells treated with cadmium or nifedipine (*p* = 0.84 and 0.27, respectively). Therefore, while the rapid glucocorticoid-induced endocannabinoid release is sensitive to nominally 0 extracellular calcium, it does not depend on calcium influx through voltage-gated calcium channels.

Endocannabinoid release is induced by GPCRs coupled to PLC activation and, consistent with this, there are cases where endocannabinoid synthesis requires calcium release from intracellular stores (Hashimotodani et al., 2005; Melis et al., 2004; Robbe et al., 2002; van der Stelt and Di Marzo, 2005). To test for a requirement for calcium release from intracellular stores, the calcium ATPase pump inhibitor thapsigargin was introduced into the recorded cells via the patch pipette for 30 min after establishing the whole-cell configuration and prior to the bath application of Dex (1–10 μM). In the presence of intracellular thapsigargin (5 μM), the Dex-induced suppression of mEPSC frequency was abolished (*p* = 0.40, *n* = 10) (Fig. 4C). Because intracellular calcium release can be mediated by activation of IP3 receptors, we also tested for an IP3 receptor dependence of the glucocorticoid effect with the IP3 receptor antagonist Xestospongin C applied via the patch pipette. In the presence of intracellular Xestospongin C (1 μM), the effect of Dex (1 μM) on mEPSC frequency was again abolished (*p* = 0.87, *n* = 5) (Fig. 4C). A between-group comparison revealed a significant difference between Dex alone and Dex in the presence of Xestospongin C on mEPSC frequency (*p* < 0.05, unpaired *t*-test), and a trend toward a difference between Dex alone and Dex in the presence of thapsigargin (*p* = 0.087). These results together suggest that calcium release from intracellular stores via activation of IP3 receptors is required for the glucocorticoid-induced endocannabinoid suppression of synaptic excitation.

### 3.7 PLC activation is sufficient to cause endocannabinoid release

**Fig. 5:**
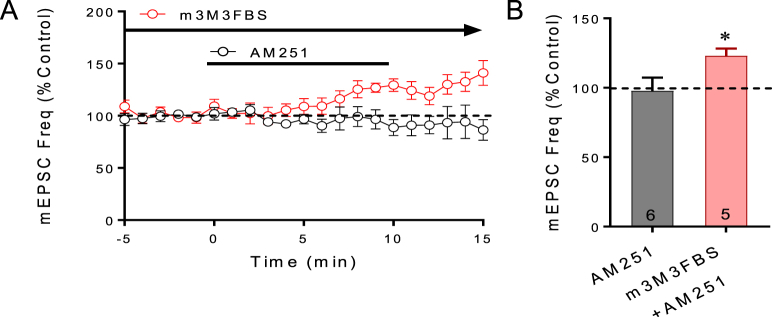
**PLC activation stimulates endocannabinoid release.** A. Running averages of mEPSC frequency with or without intracellular activation of PLC. With a normal patch solution, the CB1 receptor antagonist AM251 had little effect on the mEPSC frequency. With intracellular application of the PLC activator m3M3FBS via the patch pipette, AM251 caused an increase in mEPSC frequency. B. Summary bar graph of the changes in the mean normalized mEPSC frequency in response to CB1 blockade with AM251 with (m3M3FBS + AM251) and without (AM251) intracellular application of the PLC activator. *, *p* < 0.05.

2-AG synthesis has been shown to be stimulated in neurons by G protein-coupled receptor activation of phospholipase C (PLC), which may be facilitated by convergent calcium signaling (Maejima et al., 2005). Phospholipase C canonically activates PKC and ERK in a calcium-dependent manner, thus incorporating all three signals. Therefore, we applied a PLC activator, m3M3FBS, via the patch pipette to activate postsynaptic PLC in order to determine whether PLC stimulates endocannabinoid release. As with intracellular application of the PKA activator, following >10 min of intracellular application of m3M3FBS, the CB1 antagonist AM251 was then applied in the bath perfusion to test for an increase in mEPSC frequency, which will indicate an m3M3FBS-induced tonic suppression of mEPSCs mediated by endocannabinoid release. In cells in which m3M3FBS (10 μM) was applied intracellularly, AM251 (25 μM) caused a 25% increase in the mEPSC frequency (*p* < 0.05, n = 6) (Fig. 5), indicating a PLC-induced release of endocannabinoid. Activation of PLC, therefore, is sufficient to mimic the glucocorticoid-induced suppression of synaptic excitation, implicating PLC as a likely signal upstream of PKC, ERK and calcium activation in the membrane glucocorticoid receptor signaling pathway.

## 4 Discussion

**Fig. 6:**
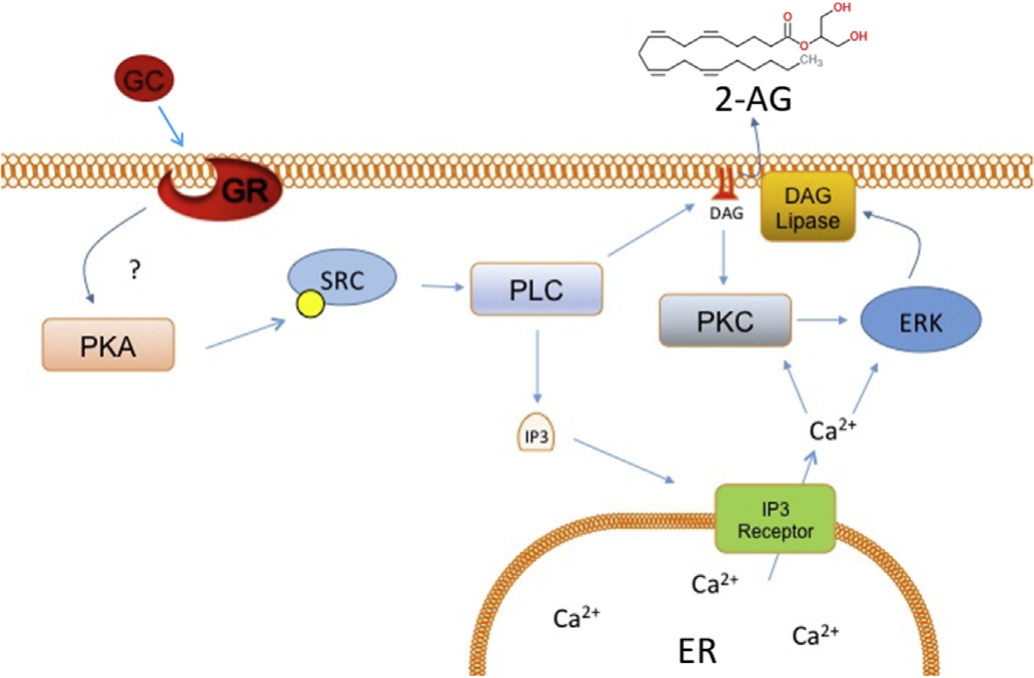
**Model of the signaling pathway for membrane glucocorticoid receptor-induced endocannabinoid synthesis.** The membrane-associated glucocorticoid receptor (GR) may be either the nuclear glucocorticoid receptor located at the membrane or an as-yet unidentified G protein-coupled receptor. Binding of glucocorticoid (GC) to the membrane GR triggers activation of PKA early in the pathway, upstream from PKC and ERK-MAPK, presumably via a Gαs-dependent mechanism (Malcher-Lopes et al., 2006). PKA then activates a series of signaling molecules, including Src, PLC, PKC, IP3 receptors, and ERK-MAP kinase. Our data suggest that activation of PLC occurs without upstream activation of Gq, and that this leads to IP3 and PKC production. The glucocorticoid-induced endocannabinoid release also requires IP3 receptor activation and store calcium mobilization. The endocannabinoid synthesized by glucocorticoid activation of a membrane receptor is likely to be 2-AG.

Here, we present evidence from pharmacological analyses in brain slices of a complex signaling pathway downstream from a membrane glucocorticoid receptor that stimulates the synthesis and release of the endocannabinoid 2-AG, which leads to the suppression of excitatory synaptic transmission in hypothalamic magnocellular neurons. A working model of the membrane glucocorticoid receptor signaling pathway is presented in Fig. 6. Magnocellular neurons were identified based on electrophysiological hallmarks (Tasker and Dudek, 1991; Luther and Tasker, 2000), but we did not distinguish between oxytocin and vasopressin magnocellular neurons because both cell types respond with a qualitatively similar response to glucocorticoid (Di et al., 2003); we worked under the assumption, therefore, that the signaling pathways are the same in both cell types. We found no evidence in these studies for a bimodal distribution in the data that would suggest different signaling mechanisms in the two cell types. Glucocorticoid suppression of synaptic excitation in both cell types, therefore, should cause a negative feedback inhibition of the stress-induced secretion of both oxytocin and vasopressin. While lack of cell-type specificity would not allow for differential modulation of the two neurohormones during their simultaneous release, situationally relevant regulation would be achieved under conditions of preferential activation of one or the other of the two hormones. For example, nursing causes oxytocin release, but also induces a rise in blood levels of corticosterone (Fehér et al., 2010; Walker et al., 1992), which could contribute to the intermittent nature of the activation of oxytocin neurons that is characteristic of the milk ejection reflex. Similarly, glucocorticoid feedback inhibition of vasopressin release increases the rate of survival in response to hemorrhage by preventing the depletion of vasopressin from prolonged activation of the vasopressin neurons (Darlington et al., 1990).

### 4.1 Glucocorticoids suppress glutamate release by releasing 2-AG

We found that the rapid glucocorticoid-induced suppression of glutamate release is abolished by blocking 2-AG synthesis in magnocellular neurons. These data are consistent with findings from other studies that implicate 2-AG as the main endocannabinoid responsible for depolarization- and G protein-coupled receptor-induced endocannabinoid signaling in the brain (Best and Regehr, 2010; Hashimotodani et al., 2005; Stella and Piomelli, 2001; Tanimura et al., 2010). Previous findings in magnocellular neurons indicate that AEA is released tonically at GABA synapses (Oliet et al., 2007) and 2-AG release is induced at glutamate synapses in response to glucocorticoid and depolarization (Di et al., 2013). While both AEA and 2-AG content increases in PVN and SON tissue samples in response to glucocorticoid application, the AEA level is an order of magnitude lower than the 2-AG level (Malcher-Lopes et al., 2006). We tested two different DAGL inhibitors to block 2-AG synthesis, both of which abolished the Dex effect completely. These data suggest, therefore, that glucocorticoids induce 2-AG synthesis at glutamate synapses on magnocellular neurons, which is consistent with our previous findings (Di et al., 2013).

### 4.2 PKA is necessary and sufficient for endocannabinoid release in magnocellular neurons

PKA is required for glucocorticoid-induced endocannabinoid release from magnocellular neurons because the rapid glucocorticoid effects were abolished by an antibody against Gαs and by PKA antagonists (Malcher-Lopes et al., 2006). Here, we show that PKA is also sufficient for endocannabinoid release because intracellular PKA activation induced a CB1 receptor-dependent decrease in the frequency of mEPSCs. PKA has been shown to directly phosphorylate DAG lipase purified from brain microsomes (Rosenberger et al., 2007), although direct DAG lipase phosphorylation by PKA is unlikely to be at play here because the PKA activation of endocannabinoid release in magnocellular neurons was PKC-dependent. 2-AG synthesis was also found to be dependent on both PKA and PKC activities in sensory neurons, although this was calcium-independent (Vellani et al., 2008).

### 4.3 PKC activity is necessary for glucocorticoid-induced endocannabinoid release

Previously, we found that PKC is required for rapid glucocorticoid-induced retrograde endocannabinoid suppression of synaptic excitation in magnocellular neurons (Di et al., 2003). Here, we showed that a broad-spectrum, membrane-permeant PKC inhibitor applied to the bath blocked the glucocorticoid effect, but left the presynaptic CB1 signaling intact, and that a membrane-impermeant PKC inhibitor applied directly within the postsynaptic neuron also blocked the glucocorticoid-induced decrease in mEPSC frequency. Together, these findings implicate PKC in the signaling pathway of the membrane glucocorticoid receptor in magnocellular neurons. Another study in sensory neurons found that thrombin, an inflammatory mediator, caused 2-AG and AEA release by activating protease-activated receptors, which are linked to the PLC/IP3/PKC pathway, and that the effect of thrombin was mimicked with PKA and PKC activators and blocked with a specific PKCε inhibitor (De Petrocellis et al., 2008).

Different PKC isoforms target different subcellular substrates. PKC also plays a role in the metabolism of DAG, the precursor of 2-AG, via its interaction with DAG kinase. The enzyme DAG kinase phosphorylates DAG, thus converting it to phosphatidic acid. PKC is able to inhibit DAG kinase activity via phosphorylation and augment DAG concentration to indirectly promote 2-AG synthesis (Luo et al., 2003).

### 4.4 Sequential PKA and PKC signaling in glucocorticoid-induced endocannabinoid release

We have established that PKA and PKC act postsynaptically to contribute to endocannabinoid release, yet, unlike PKA, we found that PKC, while necessary, was not sufficient by itself to induce endocannabinoid release. PKC inhibition blocked the PKA-induced endocannabinoid release, which we interpret to mean that PKC is required downstream of PKA and that they interact sequentially to induce endocannabinoid release (Fig. 6). We showed previously that Gαs activity, but not Gαq/11 activity, is necessary for rapid glucocorticoid-induced endocannabinoid release (Malcher-Lopes et al., 2006), which suggests that the glucocorticoid-induced PKC activity is not mediated by activation of a Gαq-coupled receptor. Previously it has been shown that PKA sequentially activates PLC in cultured dopaminergic neurons and in proximal renal tubules (Graness et al., 1997; Han et al., 2007; Liu and Simon, 1996; Yao et al., 2008). We found that PLC activation with m3M3FBS was sufficient to activate endocannabinoid synthesis. Since PKC is activated canonically by PLC production of DAG and IP3, and since the glucocorticoid effect was blocked by blocking IP3 receptors, this suggests that PLC and PKC are downstream from PKA in the pathway (Fig. 6). Nevertheless, we cannot completely rule out the possibility that PKC is activated in parallel with PKA and serves a permissive role in the signaling pathway from the membrane glucocorticoid receptor in magnocellular neurons. Also, the DAG produced by PLC may serve as the substrate for 2-AG synthesis.

### 4.5 Calcium dependence of glucocorticoid-induced endocannabinoid release

We found that the rapid glucocorticoid-induced, 2-AG-dependent suppression of glutamate release was blocked by the intracellular calcium chelator BAPTA, intracellular calcium store depletion, and antagonists of the IP3 receptor, but not by blocking voltage-gated calcium channels, which suggests that the endocannabinoid synthesis is dependent on IP3 receptor-mediated calcium release from intracellular stores. Actions downstream from a membrane glucocorticoid receptor have been reported that depend on the modulation of calcium influx (ffrench-Mullen, 1995; He et al., 2003), but this, to our knowledge, is the first report of the dependence of rapid glucocorticoid signaling on calcium release from intracellular stores. The glucocorticoid-induced endocannabinoid signaling was also blocked by depletion of extracellular calcium, which suggests that the extracellular calcium concentration acts indirectly on endocannabinoid synthesis by altering the intracellular calcium equilibrium. However, calcium entry other than via voltage-gated calcium channels may be responsible for the dependence of the glucocorticoid effect on extracellular calcium, since magnocellular neurons express ionotropic purinergic receptors, osmosensitive TRPV cation channels, and calcium-permeable NMDA receptors that provide alternative sources of calcium influx (Chakfe and Bourque, 2001; Panatier, 2009; Song et al., 2010).

### 4.6 Proposed signaling pathway for glucocorticoid induced endocannabinoid synthesis

Activation of Gs and PKA are likely to be early signals in the glucocorticoid-induced endocannabinoid synthesis pathway (Malcher-Lopes et al., 2006). Pharmacological activation of PKA was sufficient for endocannabinoid production, which was blocked by inhibiting both PKC and ERK. Since PLC is canonically upstream of PKC, ERK and intracellular calcium mobilization, all signals implicated in the endocannabinoid response, it is also likely an early signal. PKA has conversely been reported to inhibit PKC pathways in other systems (Rahamim Ben-Navi et al., 2016), but cooperative cross-talk between these signaling pathways has also been observed (Gomes and Soares-Da-Silva, 2012; Yao et al., 2008). Src kinase could be acting as an intermediary to allow the PKA signal to activate PLC and, successively, PKC and ERK. Src has been shown to be a phosphorylation target for PKA (Obara et al., 2004) and can complex with and activate PLC (Zachos et al., 2013). Src involvement could also implicate rapid signaling via the nuclear GR, as Src is associated with HSP90 and is activated upon dissociation from the ligand-bound GR (Samarasinghe et al., 2011). Our proposed signaling pathway, therefore, consists of activation of PKA, followed by Src, followed by PLC. PLC then produces IP3, which causes intracellular calcium release, and DAG, which activates PKC and ERK. Calcium and ERK signaling then cause production of 2-AG, which may be supplemented by the increase in available DAG. While we do not have direct evidence for interaction between the steps in our proposed signaling pathway, this signaling sequence provides a plausible working model for future, finer-grained studies of glucocorticoid signaling to endocannabinoid synthesis via a membrane-associated glucocorticoid receptor.

The most vexing piece of this pathway that is missing is the membrane-associated glucocorticoid receptor. While it is clear that glucocorticoids must be interacting with a membrane receptor in hypothalamic neuroendocrine cells (Di et al., 2013, 2009; 2003; Malcher-Lopes et al., 2006; Nahar et al., 2016), identification of the membrane glucocorticoid receptor has proven elusive. A G protein-coupled receptor (GPCR) is a possible candidate, as blocking G proteins, including Gs but not Gq, blocks glucocorticoid-induced endocannabinoid synthesis, and our current and past findings suggest GPCR signaling mechanisms (Di et al., 2013, 2009; 2003; Malcher-Lopes et al., 2006; Nahar et al., 2016). The abundance of orphan GPCR's (Ngo et al., 2016) allows for the possibility of an undiscovered G protein-coupled glucocorticoid receptor, as was found for estrogen (Filardo and Thomas, 2012; Qiu et al., 2008). Alternatively, glucocorticoids could be acting indirectly on known GPCR's involved in other signaling processes, similar to the interaction between estrogen and metabotropic glutamate receptors (Meitzen and Mermelstein, 2011; Micevych and Mermelstein, 2008). Yet another possibility is that the rapid glucocorticoid actions could be mediated by biased GPCR signaling via β-arrestin linked to ERK signaling (Luttrell and Lefkowitz, 2002), which is consistent with the potential involvement of multiple signaling molecules, as we report here. We have also shown that a membrane glucocorticoid receptor signals to the nuclear glucocorticoid receptor in hypothalamic neurons to stimulate the translocation of the unliganded receptor to the nucleus (Rainville et al., 2017). Finally, independent of GPCRs, the nuclear glucocorticoid receptor could act at or near the membrane by initiating signaling alternate to traditional transcriptional regulation. The nuclear glucocorticoid receptor has been located at the membrane in hypothalamic (Liposits and Bohn, 1993; Nahar et al., 2016) and other neurons (Johnson et al., 2005; Nicolaides et al., 2017; Shaqura et al., 2016), and knocking out the nuclear glucocorticoid receptor in hypothalamic neurons abolishes rapid glucocorticoid-induced endocannabinoid modulation of excitatory transmission (Nahar et al., 2016). However, GR knockouts will have a drastically altered transcription profile, confounding the knockout phenotype as evidence for a direct role of GR in endocannabinoid synthesis. Determining the early membrane-associated signals in the glucocorticoid rapid-action pathway would be invaluable for finally determining how glucocorticoids interact with the membrane.
